# Drug-Repositioning Screening for Keap1-Nrf2 Binding Inhibitors using Fluorescence Correlation Spectroscopy

**DOI:** 10.1038/s41598-017-04233-3

**Published:** 2017-06-21

**Authors:** Yuki Yoshizaki, Takayasu Mori, Mari Ishigami-Yuasa, Eriko Kikuchi, Daiei Takahashi, Moko Zeniya, Naohiro Nomura, Yutaro Mori, Yuya Araki, Fumiaki Ando, Shintaro Mandai, Yuri Kasagi, Yohei Arai, Emi Sasaki, Sayaka Yoshida, Hiroyuki Kagechika, Tatemitsu Rai, Shinichi Uchida, Eisei Sohara

**Affiliations:** 1 Department of Nephrology, Graduate School of Medical and Dental Sciences, Tokyo Medical and Dental University, Tokyo, Japan; 2 Chemical Biology Screening Center and Department of Medicinal and Organic Chemistry, Institute of Biomaterials and Bioengineering, Tokyo Medical and Dental University, Tokyo, Japan

## Abstract

The Kelch-like ECH-associating protein 1 (Keap1)-nuclear factor erythroid 2-related factor 2 (Nrf2)-antioxidant response element (ARE) signaling pathway is the major regulator of cytoprotective responses to oxidative and electrophilic stress. The Cul3/Keap1 E3 ubiquitin ligase complex interacts with Nrf2, leading to Nrf2 ubiquitination and degradation. In this study, we focused on the disruption of the Keap1-Nrf2 interaction to upregulate Nrf2 expression and the transcription of ARE-controlled cytoprotective oxidative stress response enzymes, such as HO-1. We completed a drug-repositioning screening for inhibitors of Keap1-Nrf2 protein-protein interactions using a newly established fluorescence correlation spectroscopy (FCS) screening system. The binding reaction between Nrf2 and Keap1 was successfully detected with a K_D_ of 2.6 μM using our FCS system. The initial screening of 1,633 drugs resulted in 12 candidate drugs. Among them, 2 drugs significantly increased Nrf2 protein levels in HepG2 cells. These two promising drugs also upregulated ARE gene promoter activity and increased HO-1 mRNA expression, which confirms their ability to dissociate Nrf2 and Keap1. Thus, drug-repositioning screening for Keap1-Nrf2 binding inhibitors using FCS enabled us to find two promising known drugs that can induce the activation of the Nrf2-ARE pathway.

## Introduction

Oxidative stress occurs as a result of increased reactive oxygen species (ROS) and/or depressed capacity of the antioxidant system^1^. The transcription factor, nuclear factor erythroid 2-related factor 2 (Nrf2), plays a key role in cellular antioxidant defenses and maintains redox homeostasis. Under normal conditions, Nrf2 is retained in the cytoplasm by Keap1 and constantly subjected to ubiquitination and degradation mediated by the binding of Keap1 to the Cul3/Rbx1 E3 ubiquitin ligase complex^2^. When the complex is exposed to oxidative/electrophilic stress, Nrf2 is released from Keap1 and translocates into the nucleus. Nrf2 binds to the antioxidant response element (ARE) in the promotor region of a wide range of antioxidant and detoxifying enzymes^3^, such as heme oxygenase-1 (HO-1)^4^. There are many experimental reports demonstrating that enhancement of Nrf2 function is a promising antioxidant strategy and can be especially effective in treating broadly-defined inflammatory diseases. For example, it was reported that myeloid-derived Nrf2 activity attenuates atherosclerosis development, liver inflammation, and fibrosis associated with obesity in an obese hypercholesterolemic mice model^5^. In terms of rheumatoid arthritis, Wruck *et al*. showed that oxidative stress is significantly involved in cartilage degradation in experimental arthritis, and the presence of a functional Nrf2 gene is a major requirement for limiting cartilage destruction^6^. Khor *et al*. reported that Nrf2 contributed to intestinal protection through regulation of proinflammatory cytokines and induction of phase II detoxifying enzymes using mice model with dextran sulfate sodium-induced experimental colitits^7^, suggesting the usefulness of Nrf2 activation for inflammatory bowel disease. Other than these, clinical usefulness for type I diabetes^8, 9^, HIV^10, 11^, and neurological disorders such as Parkinson’s disease^12, 13^ has been also presented. In addition, in terms of preventive medicine, activation of Nrf2 signaling pathway is considered as a promising strategy for cancer chemoprevention, leading to the expression of cytoprotective genes against cancer. In general, these reports support the idea that the activation of Nrf2 could contribute to prevention of numerous diseases. Recent studies have shown that several natural products, including sulforaphane, Glycyrrhiza glabra, resveratrol, curcumin, and epigallocatechin-3-gallate have the potential as an Nrf2 activator with cytoprotective functions^14–18^. These are also expected as potential Nrf2 activators.

Inhibition of Keap1-Nrf2 protein-protein interaction (PPI) is a promising strategy to promote Nrf2 activation^19^. Indeed, several reports showed the usefulness of modifying protein-protein interactions as a therapeutic target for Nrf2 signaling^20^. Recently, we reported the identification of promising compounds that may disrupt PPIs using fluorescence correlation spectroscopy (FCS)^21^. Our present study aimed to discover novel Keap1-Nrf2 binding inhibitors using FCS. Furthermore, we focused on “drug repositioning”, i.e., the application of known drugs to treat new indications, in an attempt to reduce costs and risks.

## Results

### Detection of Keap1-Nrf2 binding using FCS

Nrf2 possesses 6 highly conserved domains known as Nrf2-ECH homology (Neh) domains. Of these, the Neh2 domain localized in the N-terminal region is essential for the Keap1-dependent degradation of Nrf2^22–24^. Furthermore, this domain directly associates with the β-barrel structure of Keap1, which contains a DGR domain with 6 Kelch repeats^25^. The Neh2 domain harbors two different Keap1-binding motifs: DLG with low-affinity (^23^LWRQDIDLGVSREVF^37^) and ETGE (^76^LDEETGEFLP^85^) with high-affinity^25^. Both binding domains play a crucial role in the interaction with homodimerized Keap1 via a hinge-and-latch mechanism^26^. We focused on identified chemical compounds that efficiently disrupted the binding of the ETGE motif in Nrf2 to the DGR domain in Keap1. We established a system capable of high-throughput screening to detect the binding of two molecules using FCS^21^. Briefly, FCS is a method capable of measuring the fluctuation rate of a fluorescently-labeled single peptide with an output of the translational diffusion times. When a fluorescently-labeled molecule binds to another molecule, the translational diffusion time is extended by increasing the apparent molecular mass^27^. According to this principle, we first determined if we could detect the binding of Keap1 to Nrf2. Fluorescent TAMRA-labeled small peptides containing the ETGE (LDEETGEFLP) motif of Nrf2 were mixed with GST-fusion proteins of the DGR domain of Keap1 (GST-Keap1-DGR) at various concentrations (3.7 × 10^1^–7.5 × 10^4^ nM) in 384-well microtiter plates. The translational diffusion time in each well was measured using FluoroPoint-Light (Olympus). The addition of GST-Keap1-DGR dose-dependently increased the translational diffusion time of Nrf2-ETGE, indicating that this method efficiently detects the binding of Keap1 to Nrf2. The association/binding curve between Nrf2 and GST-Keap1-DGR obtained in the FCS assay is shown in Supplementary Fig. S1. The K_D_ value for Keap1-Nrf2 binding was 2.6 μM.

### Drug-repositioning screening for compounds that potentially disrupt Keap1-Nrf2 binding using FCS

**Figure 1:**
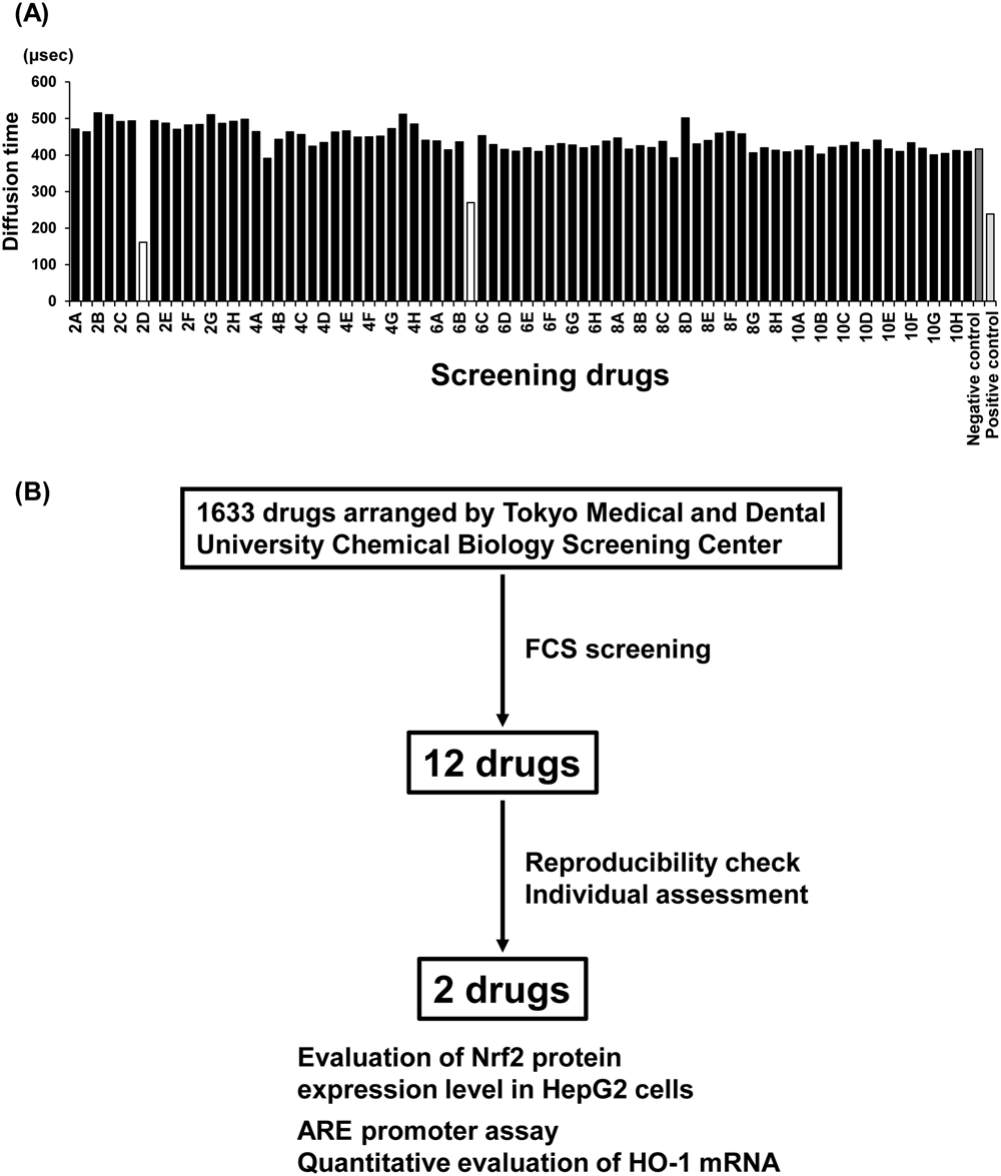
High-throughput screening of Keap1-Nrf2-binding inhibitors. (**A**) Representative results of the FCS screening are shown. Known drugs in our chemical library were added individually to the mixed solution of Nrf2-ETGE and GST-Keap1-DGR. Nrf2-ETGE and GST-Keap1-DGR without the addition of test compounds was used as the negative control (NC, solvent control for binding state), and Nrf2-ETGE and GST-alone was used as the positive control (PC, dissociation control). White bars show the drugs with potential inhibitory effects on binding. (**B**) Flow chart of this drug screening project.

We used this system to screen 1,633 known drugs owned by the Tokyo Medical and Dental University Chemical Biology Screening Center. The representative results of the initial screening are shown in Fig. 1A. Although most of the compounds hardly exhibited inhibitory binding effects, a few compounds restored the diffusion time to baseline values, suggesting that these compounds cause the dissociation of Keap1 and Nrf2. The initial screening of 1,633 drugs revealed 12 drugs that reproducibly showed inhibitory effects on Keap1-Nrf2 binding using FCS (Fig. 1B, see Supplementary Fig. S2), even though apparent structural similarities among the 12 drugs were not found.

### Two drugs increased the protein expression of Nrf2 in HepG2 cells

**Figure 2:**
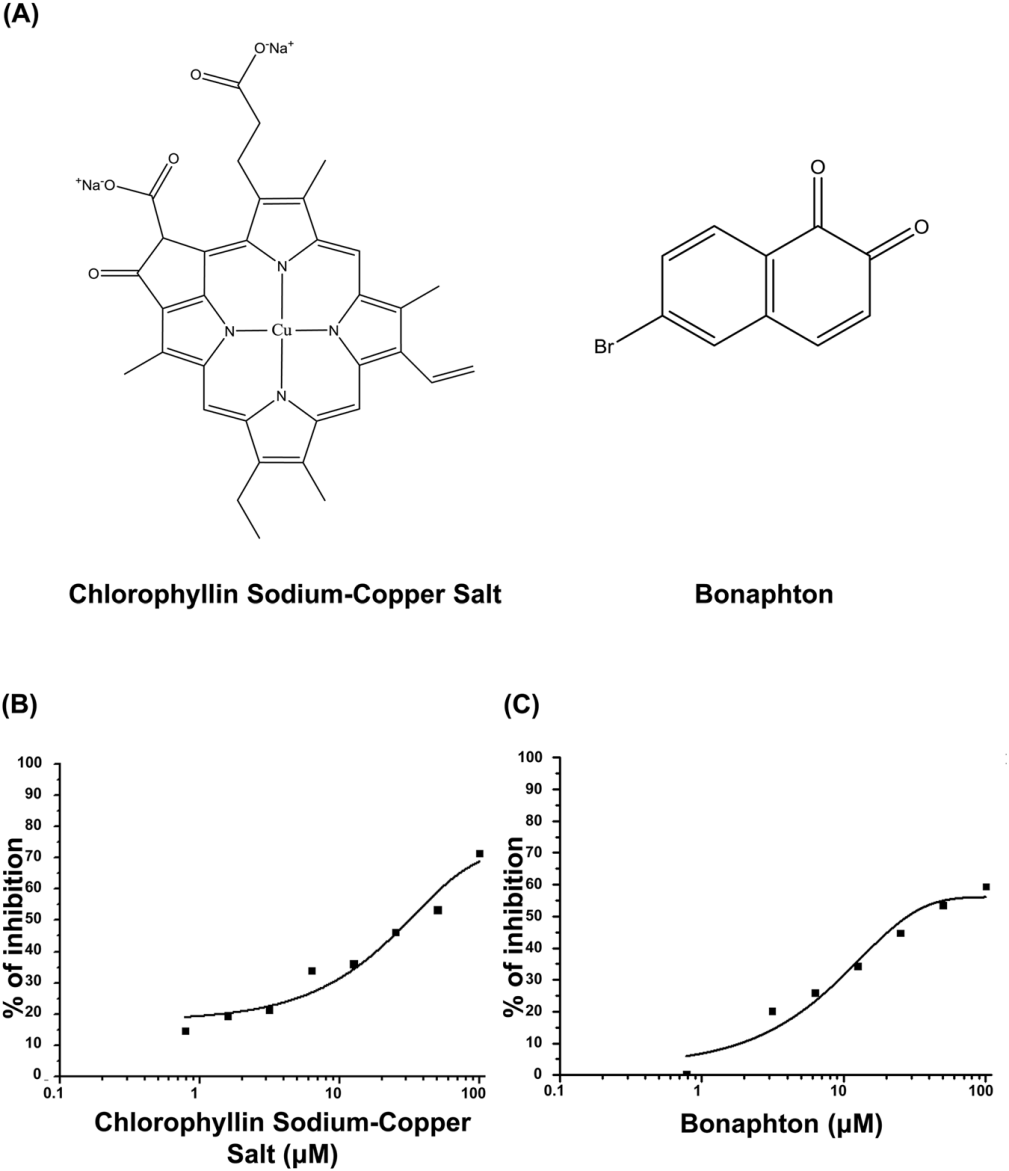
Two promising drugs obtained by FCS screening. (**A**) Chemical structures and names of the two candidate drugs: chlorophyllin sodium copper salt and bonaphton. (**B**) and (**C**) Binding inhibition curve based on the drug concentration. The two drugs were independently added to a mixed solution of GST-Keap1-DGR and Nrf2-ETGE at various concentrations (0.78–100 μM). The IC_50_ values of chlorophyllin sodium copper salt and bonaphton were 35.7 and 37.9 μM, respectively.

**Figure 3:**
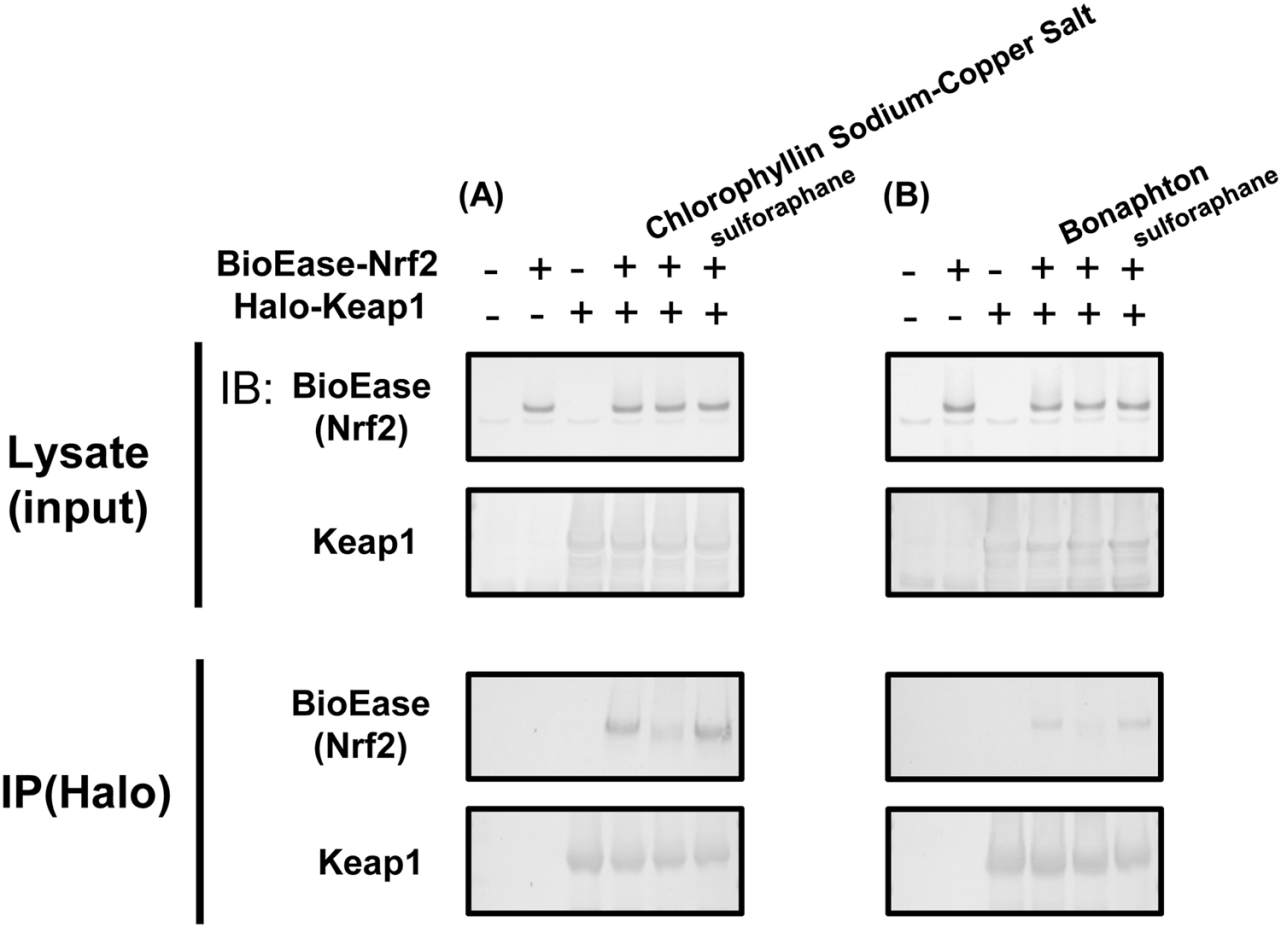
Effect of chlorophyllin sodium copper salt and bonaphton on binding of Keap1 to Nrf2. To clarify that (**A**) chlorophyllin sodium copper salt, and (**B**) bonaphton disrupt the interaction between Keap1 and Nrf2 by an alternative experiment other than FCS, we performed co-immunoprecipitation assay for the verification of disruption of Keap1 interaction by these compounds. BioEase-tagged Nrf2 was successfully immunoprecipitated by Halo-tagged Keap1, using Halo-antibody. Both of the compounds induced consistent decrease in binding between the two proteins, although protein-protein interaction between Keap1 and Nrf2 was not decreased by sulforaphane that does not disrupt interaction between Keap1 and Nrf2. This result indicated that these compounds inhibited the Keap1-Nrf2 protein-protein interactions directly. Experiments were repeated three times with consistent results, and a representative blot is shown. Full-length blots are presented in Supplementary Fig. S3.

**Figure 4:**
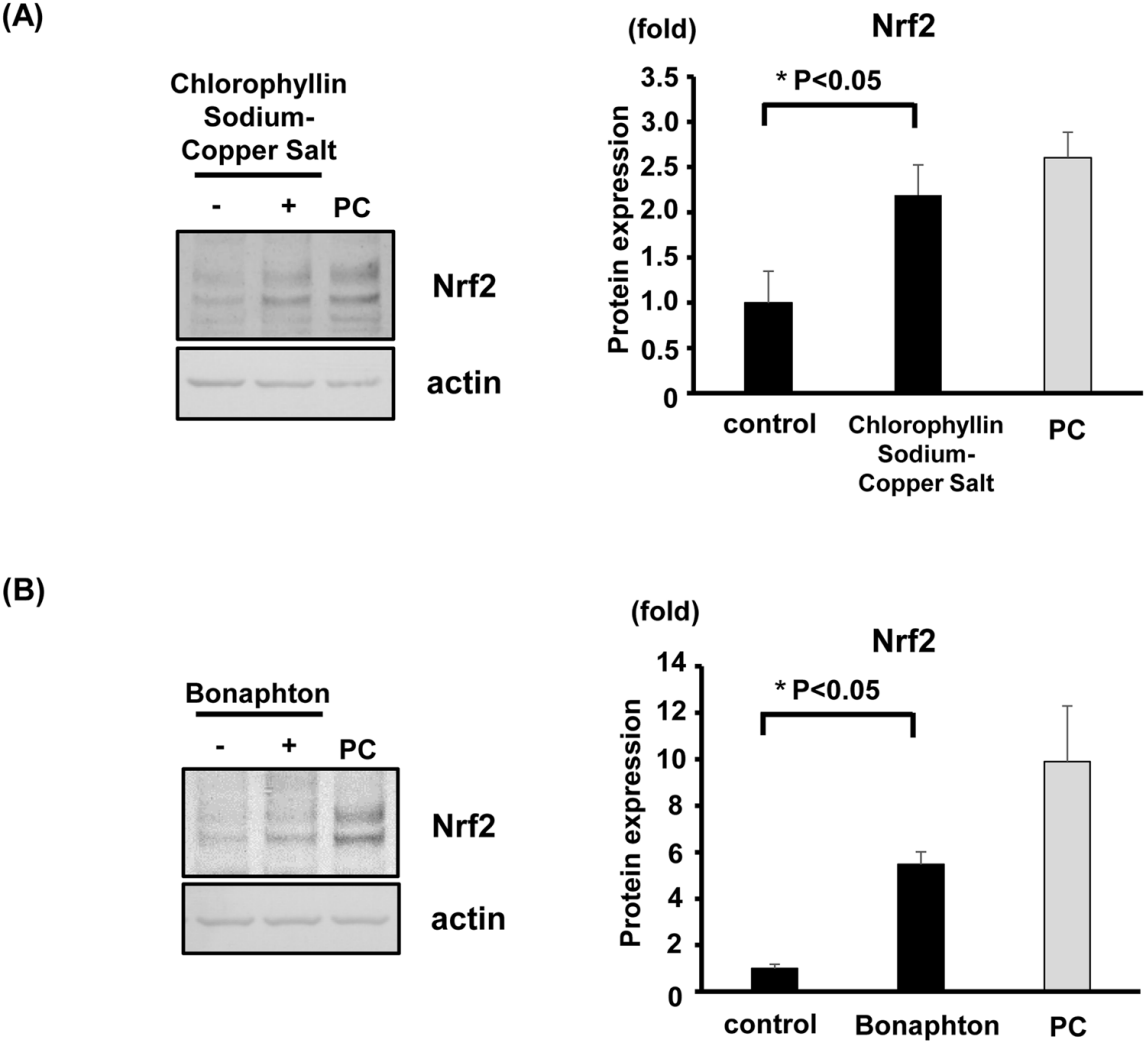
Nrf2 protein expression in HepG2 cells is enhanced by chlorophyllin sodium copper salt and bonaphton. HepG2 cells were treated with the two compounds for 5 h. Sulforaphane, a known Nrf2 activator, was used as a positive control (5 μM; PC). Total cell lysates were immunoblotted using the Nrf2 antibody. (**A**) Chlorophyllin sodium copper salt (100 μM) increased Nrf2 protein expression. (**B**) Bonaphton (100 μM) increased Nrf2 protein expression. Values are presented as the mean ± S.E.M. *p < 0.05 vs. vehicle-treated cells, t-test (n = 4 per experimental group). Experiments were repeated four times with consistent results, and a representative blot is shown. Full-length blots are presented in Supplementary Fig. S4.

**Figure 5:**
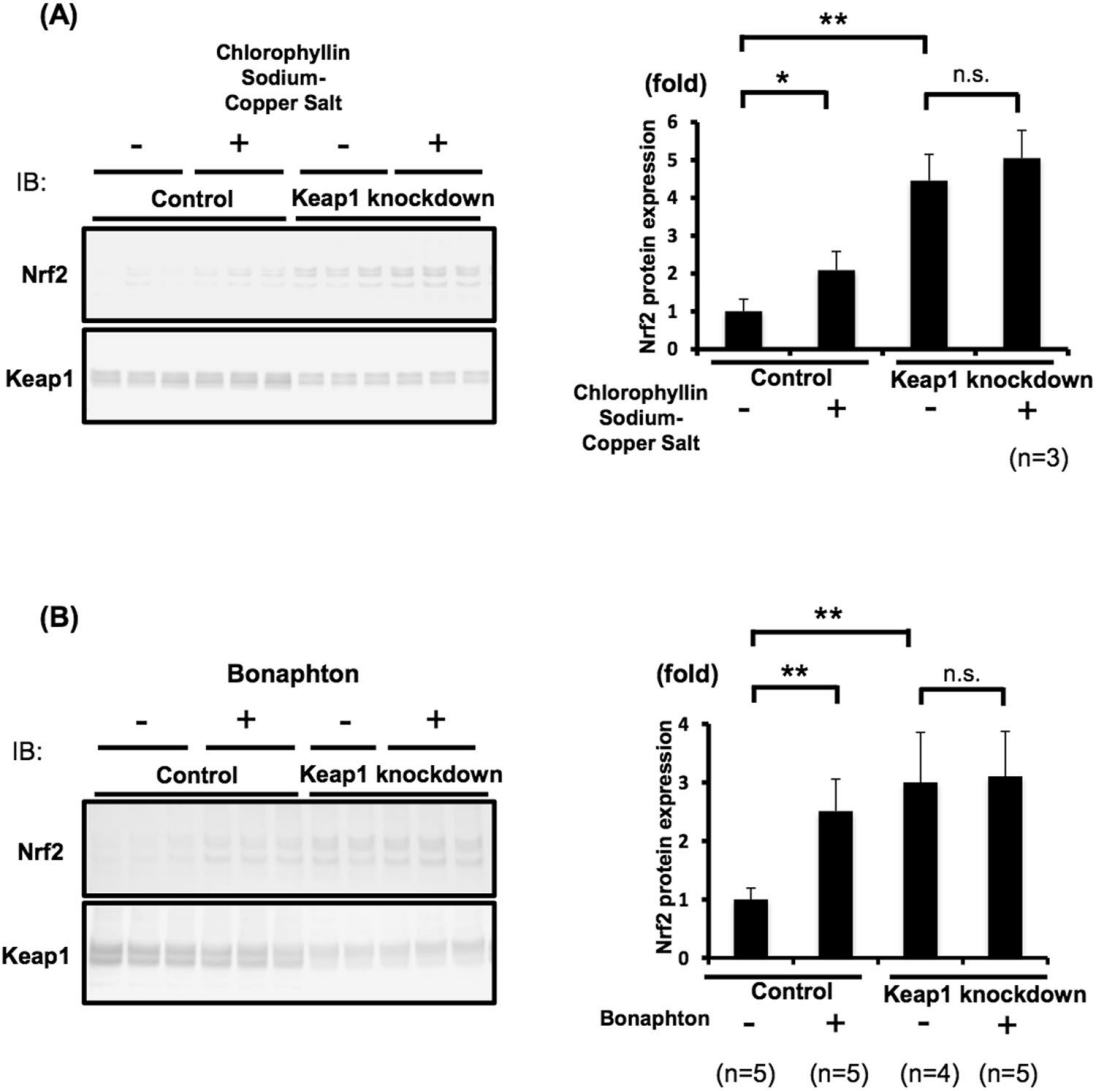
The effects of chlorophyllin sodium copper salt and bonaphton are depending on Keap1. HepG2 cells transfected with siRNA for Keap1 were treated with the two compounds. Protein expression levels of Keap1 were decreased by knock down, leading to increases of Nrf2 expression. The increases of Nrf2 by these compounds are not significant when Keap1 is knocked down, indicating that the effects of the compounds are depending on Keap1. (**A**) Left: Immunoblots of Keap1 knockdown cells treated with chlorophyllin sodium copper salt. Right: Densitometry analysis of Nrf2. (**B**) Left: Representing immunoblots of Keap1 knockdown cells treated with bonaphton. Right: Densitometry analysis of Nrf2 from total of 4–5 independent experiments. In densitometry analysis, values are expressed as the ratio to the average of the Nrf2 signals in the control group without compounds. *P < 0.05, **P < 0.01. Full-length blots are presented in Supplementary Fig. S5.

Out of the 12 candidates obtained in the initial screening, we selected the two drugs that exhibited the highest inhibitory activities in the FCS system: chlorophyllin sodium copper salt (chlorophyllide Cu complex Na salt, LKT Labs, Inc.) and bonaphton (6-bromo-1, 2-naphthoquinone, Vitas-M Laboratory) (Fig. 1B, Fig. 2A–C). The IC_50_ values of chlorophyllin sodium copper salt and bonaphton were 35.7 and 37.9 μM, respectively. To confirm that these compounds disrupt the interaction between Keap1 and Nrf2 by an alternative experiment other than FCS, we further performed co-immunoprecipitation assay that was used for the verification of disruption of Keap1 protein-protein interaction by the other drugs^28^. Thus, we immunoprecipitated Keap1 from cells and tested whether addition of the drugs could dissociate coimmunoprecipitated Nrf2, and found that the compounds induced consistent decrease in binding between the two proteins. This result indicated that the compounds indeed inhibited the Keap1-Nrf2 protein-protein interactions directly (Fig. 3). We then tested whether these drugs truly showed inhibitory effects on Keap1-Nrf2 binding using HepG2 cells, which reportedly express endogenous Nrf2 with 2 bands^29^. We used sulforaphane (LKT Labs, Inc.), a known Keap1 inhibitor capable of activating Nrf2 signaling, as a positive control^30^. Both chlorophyllin sodium copper salt and bonaphton significantly increased the protein expression of endogenous Nrf2 in HepG2 cells after direct exposure (Fig. 4). Moreover, to demonstrate that the effects of these two compounds are dependent on Keap1, we performed Keap1 knockdown experiments with siRNA in HepG2 cells. As shown in Fig. 5, although the expression level of Nrf2 is increased by these compounds in control, the increases of Nrf2 by these compounds are not significant when Keap1 is knocked down, indicating that the effects of the compounds are depending on Keap1.

### Two drugs induced ARE-dependent transcriptional activity in HepG2 cells

**Figure 6:**
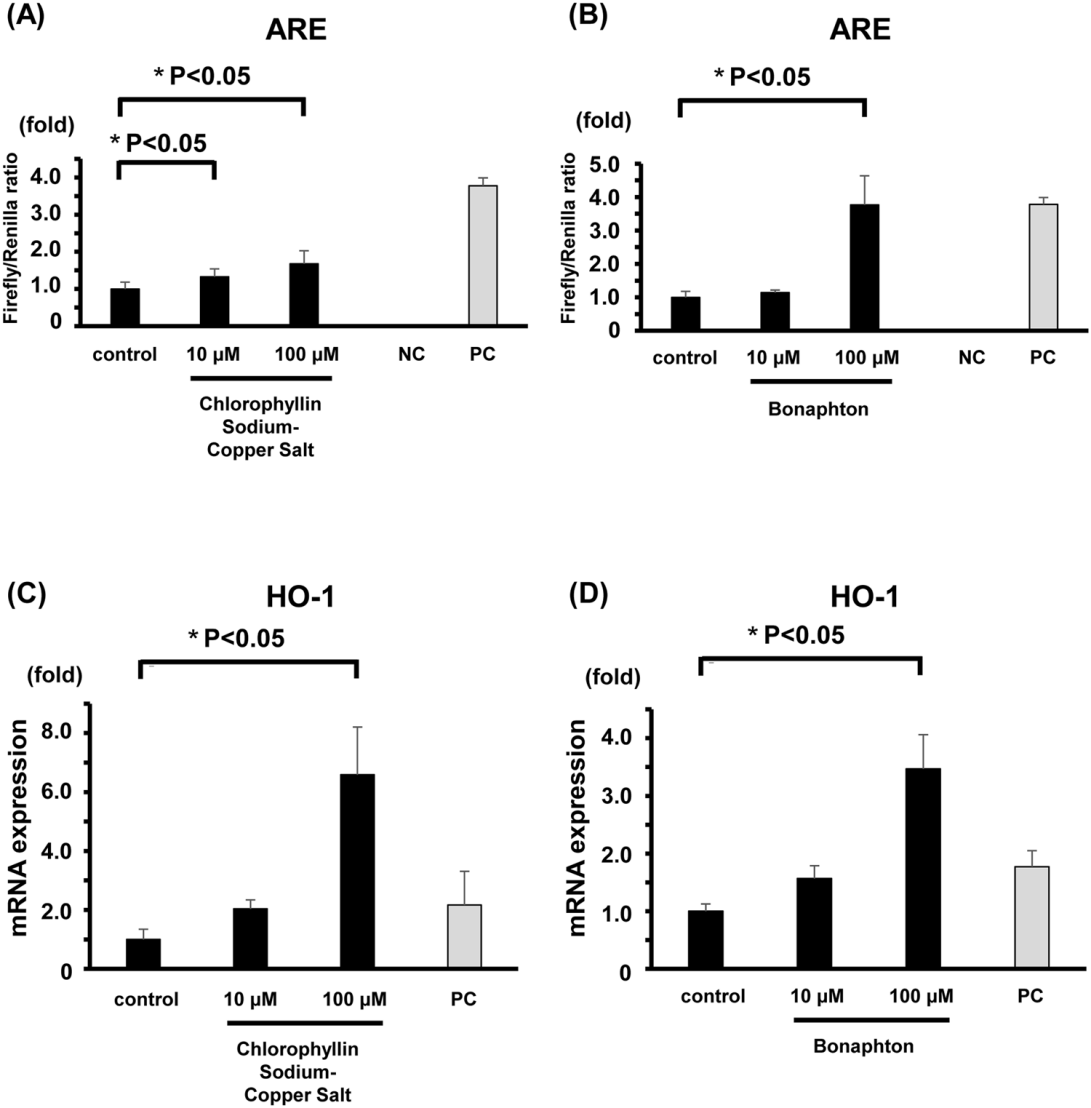
Chlorophyllin sodium copper salt and bonaphton increased ARE promoter activity and HO-1 mRNA expression in HepG2 cells. The luciferase activity of cells transfected with an ARE reporter construct were evaluated 5 h after drug exposure. (**A**) Chlorophyllin sodium copper salt at concentrations of 10 μM and 100 μM significantly activated ARE promoter activity. (**B**) Bonaphton at a concentration of 100 μM significantly activated ARE promoter activity. The negative control was a mixture of a non-inducible reporter construct and constitutively expressed Renilla luciferase construct. The positive control was a constitutively expressed GFP construct pre-mixed with constitutively expressed firefly and Renilla luciferase constructs. Data are expressed as the mean ± S.E.M. *P < 0.05 vs. vehicle-treated cells (control), t-test (n = 3 per experimental group). NC; negative control, PC; positive control. (**C**,**D**) HO-1 mRNA expression in HepG2 cells 24 h after drug treatments (10 or 100 μM) was quantified by qRT-PCR. (**C**) Chlorophyllin sodium copper salt (100 μM) significantly increased HO-1 mRNA expression. (**D**) Bonaphton (100 μM) significantly increased HO-1 mRNA expression. Five micromolar sulforaphane was used as a positive control (PC) of Nrf2 activation. Data were normalized to actin and expressed as the mean ± S.E.M. *P < 0.05 vs. vehicle-treated cells, t-test (n = 3 per experimental group).

To evaluate if chlorophyllin sodium copper salt and bonaphton exert pharmacological activities in cells, a luciferase reporter assay with ARE elements was conducted. Both chlorophyllin sodium copper salt and bonaphton (10 and 100 μM) significantly increased ARE luciferase activity (Fig. 6A and B). To further validate whether transcriptional activity of genes under the ARE promoter are activated by these candidate drugs, HO-1 mRNA expression was quantified by qRT-PCR in HepG2 cells. Both of the cells treated with chlorophyllin sodium copper salt and bonaphton increased the mRNA expression of endogenous HO-1 in a concentration-dependent manner (Fig. 6C and D). The two promising drugs obtained by screening with FCS screening induced ARE-dependent transcriptional activity in HepG2 cells likely via Keap1-Nrf2 dissociation.

## Discussion

In this study, we screened 1,633 known drugs owned by Tokyo Medical and Dental University Chemical Biology Screening Center for Keap1-Nrf2 binding inhibitors using FCS. The chemical library was mainly obtained from MicroSource Discovery Systems, Inc. (Gaylordsville, CT), which possesses approximately 2,000 compounds with known safety profiles. Most of these drugs are approved for animal or human use, and their pharmacological and toxicological profiles have been defined and published. Of the 12 candidates obtained from the initial screening, we focused on the two drugs that exhibited the highest inhibitory activity: chlorophyllin sodium copper salt and bonaphton. In our study, both drugs significantly increased ARE transcriptional activity and HO-1 mRNA expression in HepG2 cells, indicating that Nrf2 is activated via the drug-induced dissociation of Keap1 and Nrf2.

Drug repositioning is the process of identifying a new use for an existing drug or drug candidate outside the scope of the original indication^31^. This method of identification is efficient because a great deal of information, such as pre-clinical toxicology, efficacy data, and clinical pharmacokinetic properties, is already available. Chlorophyllin sodium copper salt is the most common chlorophyllin derivative, and it is mainly used as a food additive or in alternative medicine. Chlorophyllin sodium copper salt was reported to exert antiviral effects on HBV in tissue culture systems^32^. Chlorophyllin itself was already reported to exert antioxidant effects by inducing HO-1 and NQO1 expression via PI3K/Akt and Nrf2^33^. Notably, our blind screening detected the chlorophyllin derivative coincidentally, thus confirming the reliability of our screening system. Moreover, even though the previous report did not mention the pharmacological mechanism of chlorophyllin-induced Nrf2 activation, the dissociative effects of its derivative on Keap1-Nrf2 binding may be applicable here because these two compounds are structurally very similar. Bonaphton shows antiviral activity against the herpes simplex virus^34^. It was assumed that the action of bonaphton may be due to the inhibition of cytoplasmic proteins that are incorporated into the capsids, which can alter the transport of virus capsid components to the cell nucleus for assembly. In contrast, there are no previous reports on the antioxidant or organ-protective effects of bonaphton, including the modulation of Nrf2 signaling. Our results show that chlorophyllin sodium copper salt and bonaphton are promising candidates that can be safely used in the clinic.

The FCS system enables high-throughput drug screening, especially for the PPIs^21^. Compared with other methods that can detect PPIs, such as the Biacore^TM^ surface plasmon resonance (SPR) system, one of the major advantages of FCS is that immobilization of the proteins is not required. The binding reactions of each molecule can be detected directly in aqueous solution, enabling the detection of the binding under more physiological conditions. In fact, several recent reports focused on detecting a Keap1-Nrf2 binding inhibitor using fluorescence polarization (FP) assays in an aqueous solution. This method resulted in several promising compounds^19, 35^. The principles used to quantify binding and the sensitivity for binding detection are comparable between FCS and the FP assay^36^.

One of the limitations of this study is that *in vivo* validations were not performed. The IC_50_ values of chlorophyllin sodium copper salt and bonaphton calculated with FCS were 35.7 and 37.9 μM, respectively. Considering these relatively high values, further modifications of the drugs are necessary to obtain more efficient pharmacological activity. In general, the EC_50_ values in a cell based assay should be in the nanomolar range, which is true of most commercially available drugs. However, chlorophyllin sodium copper salt was reported to be safe even with extremely high doses of more than 1 gram/kg/day in rats^37^. Thus, this drug itself may exert pharmacological effects on Nrf2 signaling *in vivo* safely even with high dose administration.

In addition, we demonstrated that the binding affinity for Keap1-Nrf2 interaction was 2.6 μM by FCS. This affinity is weaker than the some reports^38, 39^. We raised two major reasons for this discrepancy. One is due to the differences in length and modification of the small peptide used in FCS. Regardless of inclusion of the common binding sequences within the small peptide of Nrf2, K_D_ reportedly differs depending on the surrounding amino acid sequences. For example, Chen *et al*. reported K_D_ was strongly affected by the peptide length^38^. Although their SPS assay showed K_D_ of 23.9 nM with H-16mer-OH Nrf2 peptide (H-AFFAQLQL*DEETGEFL*-OH; “*DEETGEFL”* shown in Italic is common sequence among the peptide used in previous reports and our peptide), K_D_ was more than 1,000 nM in case of H-8mer-OH (H-*DEETGEFL*-OH). Moreover, when N-terminus of the peptide (H-L*DEETGEFL*-OH; H-9mer-OH) is acetylated, K_D_ was improved significantly (352 nM to 21.1 nM). Inoyama *et al*. reported K_D_ of 65.1 nM with FITC-labeled 9 mer peptide by using FP assay^39^. The Nrf2 peptide we used has TAMRA in N-terminus and an extra Prolin in C-terminal in addition to the common binding motif. The other reason is the effects of dissociation speed of the molecules in FCS system. Chen *et al*. reported that the dissociation speed between Nrf2 and Keap1 is quite fast, indicating they are not stable in a binding state^38^. In contrast with the SPS assay in which the other binding molecule is fixed on a chip, both molecules are moving freely in the liquid-phase in FCS. This fact suggests that FCS may not be able to detect the bindings on Nrf2-Keap1 with high efficiency because bound molecules per unit time can decrease in FCS. We believe that these factors could explain why the binding affinity in our system is different from those in the other reports^40^.

In conclusion, we succeeded in the drug-repositioning screening of compounds for their ability to inhibit Keap1-Nrf2 binding using FCS. Our system and strategy for the discovery of PPI inhibitors for Keap1 and Nrf2 were useful; furthermore, these methods could be modified for the screening of other signaling pathways. Chlorophyllin sodium copper salt and bonaphton activate Nrf2 by inhibiting Keap1-Nrf2 protein-protein interactions. These two drugs are promising drugs with established safe clinical use. Moreover, the drug derivatives may be good candidates for new antioxidant drugs that act through Nrf2 activation.

## Materials and Methods

### Cell culture

HepG2 cells were cultured in 6-well dishes in Dulbecco’s modified Eagle’s medium (DMEM) supplemented with 10% (v/v) FBS, 2 mM L-glutamine, 100 units/ml penicillin, and 0.1 mg/ml streptomycin at 37 °C in a humidified 5% CO_2_ incubator. The cells were exposed to various concentrations of test compounds for 5 hours and then lysed with RIPA buffer containing 0.25 mM Tris/HCl (pH 8.0), 0.38% EGTA, 0.1 mM EDTA, 0.18% sodium orthovanadate, 2.1% NaF, and the complete protease inhibitor cocktail (Roche; 1 tablet per 50 ml). The protein concentrations were determined with the Bradford method^41^. After centrifugation at 12,000 *g* for 5 min at 4 °C, the supernatants were denatured for 20 min at 60 °C with SDS sample buffer (Cosmo Bio) and subjected to SDS-PAGE.

### Immunoblotting

Quantitative immunoblotting was performed as previously described^42^. Blots were probed with the following primary antibodies: anti-Nrf2 (Abcam) and anti-actin (Cytoskeleton). Alkaline phosphatase-conjugated anti-IgG antibodies (Promega) were used as secondary antibodies for immunoblotting. The intensities of the bands were analyzed and quantified using ImageJ (National Institutes of Health) software.

### Expression and purification of GST-Keap1-DGR in Escherichia coli

The DNA encoding amino acid residues 315–598 of human Keap1 (DGR domain) was cloned into a pGEX6P-1 vector. The recombinant GST-Keap1 fusion protein (GST-Keap1-DGR) was expressed in BL21 *E. coli* cells, and 1-L cultures were grown at 37 °C in 2-YT broth (1.6% (w/v) tryptone, 1% (w/v) yeast extract, and 0.5% NaCl) containing 100 µg/ml ampicillin until the attenuance at 600 nm reached 0.6. Next, 1 mM IPTG was added, and the cells were cultured for an additional 16 h at 28 °C. Cells were then isolated by centrifugation and resuspended in 40 ml of ice-cold PBS, followed by sonication (TOMY Ultrasonic Disrupters UD-201). Lysates were centrifuged at 4 °C for 5 min at 10,000 *g*. The GST-fusion proteins were affinity purified with 1.2 ml of glutathione-sepharose beads and eluted in an elution buffer containing 83 mM Tris HCl, 150 mM KOH, and 30 mM glutathione.

### FCS

Fluorescent 6-carboxytetramethylrhodamine (6-TAMRA)-labeled Nrf2 peptides containing the ETGE motif [Nrf2-ETGE (TAMRA-LDEETGEFLP)] were prepared (Hokkaido System Science). Recombinant GST-Keap1-DGR was expressed in BL21 *E. coli* cells and purified using glutathione-sepharose beads. The TAMRA-labeled Nrf2 peptides (20 nM) were incubated at room temperature (25 °C) for 30 min with various concentrations of GST-Keap1-DGR (3.7 × 10^1^−7.5 × 10^4^ nM) in a PBS with 0.05% Tween 20 reaction buffer. The FCS measurements of single-molecule fluorescence were performed using the FluoroPoint-light analytical system (Olympus)^27^. The confocal volume to detect fluorescent signals in this equipment is in the range of a single femtoliter. Therefore, the TAMRA-labeled peptide was diluted so that a single fluorophore was likely to be present in the confocal space. The final concentration of each peptide in the FCS assay was 2.5 nM. The concentration of GST-Keap1-DGR used for screening was 469 nM because the binding reaction was reproducibly detected at this concentration (see Supplementary Fig. S1). We used test compounds with the concentration of 100 μM for the initial FCS screening. The DMSO concentration used to dissolve the test compounds was 1% in the final assay solution. The assay was performed in a 384-well plate. The data acquisition time was 15 s, and the measurements were repeated five times per sample. The K_D_ and IC_50_ values were calculated using Origin 8.1 data analysis and graphing software (OriginLab).

To generate binding inhibition curves, Nrf2-ETGE and GST-Keap1-DGR without the addition of test compounds was used as the negative control (NC, solvent control for binding state), and Nrf2-ETGE and GST-alone was used as the positive control (PC, dissociation control), i.e., these controls correspond to 100% binding (NC) and 0% binding (PC). To calculate the % inhibitions, we adopted a formula as following;

$$ \% \,{\rm{of}}\,{{\rm{inhibition}}}_{{\rm{TC}}}= < 1-({{\rm{DT}}}_{{\rm{TC}}}-{{\rm{DT}}}_{{\rm{NC}}})/({{\rm{DT}}}_{{\rm{NC}}}-{{\rm{DT}}}_{{\rm{PC}}}) > \times 100( \% )$$

DT: diffusion time, TC: target concentration, NC: negative control, PC: positive control.

### Nrf2 luciferase reporter assay

The Nrf2 luciferase reporter assay was performed according to the instructions described in the Cignal Reporter Assay kit (Qiagen). HepG2 cells (4 × 10^5^ per well) were transfected with the construct containing the Nrf2 transcription response element (ARE) diluted in Opti-MEM using Lipofectamine2000 (Thermo Fisher Scientific). After 24 h of transfection, cells were treated with 100 μM chlorophyllin sodium copper salt or bonaphton for an additional 5 h. Next, luciferase activity was measured using the Dual-Glo luciferase assay system. Constitutively expressed Renilla luciferase was used as an internal control. The results are expressed as a fold induction of control cells. The negative control was a mixture of a non-inducible reporter construct and the constitutively expressed Renilla luciferase construct. The positive control was a constitutively expressed GFP construct pre-mixed with a constitutively expressed firefly luciferase construct and a constitutively expressed Renilla luciferase construct.

### Quantitative real-time (qRT)-PCR analysis of mRNA for HO-1

Total RNA was extracted using TRIzol^®^ reagent (Life Technologies) according to the manufacturer’s instructions. qRT-PCR was performed using an Thermal Cycler Dice^®^ Real Time System Lite (Takara Bio, Inc.) with SYBR^®^ Premix Ex Taq™ reagent (Takara Bio, Inc.). The forward and reverse HO-1 primers were 5′-TCTCTTGGCTGGCTTCCTTAC-3′ and 5′-GCTTTTGGAGGTTTGAGACA-3′, respectively. The actin primers were forward 5′-TGGCATTGCCGACAGGATGC-3′ and reverse 5′-TCCACACGGAGTACTTGCGC-3′. Each reaction was performed in triplicate, and the mRNA levels of HO-1 were normalized to actin.

### Keap1 knockdown in HepG2 cells

Human hepatoma cells HepG2 were cultured in 6-well dishes in Dulbecco’s modified Eagle’s medium (DMEM) supplemented with 10% (v/v) FBS, 2 mM L-glutamine, 100 units/ml penicillin, and 0.1 mg/ml streptomycin at 37 °C in a humidified 5% CO_2_ incubator. Knockdown was achieved by a chemical, transient transfection of HepG2 cells with small interfering RNA (siRNA) using the fast forward transfection method diluted in Opti-MEM using Lipofectamine RNAiMAX (Thermo Fisher Scientific). HepG2 cells were either transfected by predesigned human Keap1 siRNA or non-silencing control siRNA (Hokkaido System Science) at concentrations of 25–50 nM. Transfected and control cells were maintained for 24 hrs. Knockdown efficiency of Keap1 was determined by Western blotting.

### Co-immunoprecipitation (Co-IP) and protein analysis

HEK293T cells were transiently transfected with BioEase tagged human Nrf2 and Halo-tagged human Keap1 over-expression plasmid. The cells were treated with MG132 (25 μM, Sigm-Aldrich) and with each candidate compound at the concentration of 100 μM for two hours before harvesting. The cells were then harvested in lysis buffer composed of 50 mM Tris-HCl [pH 7.5], 150 mM NaCl, 1% NP-40, 1 mM sodium orthovanadate, 50 mM sodium fluoride, and protease inhibitor cocktail, for 30 min at 4 °C. After centrifugation at 15,000 g for 10 min, the supernatant was used for immunoprecipitation by anti-Halo beads (Halo Link Resin, Promega) for 6 h at 4 °C, with/without candidate compounds at the concentration of 100 μM. Thereafter, the beads were washed three times with PBS and the lysis buffer, and the immunoprecipitates were eluted by boiling for 20 min at 60 °C in SDS sample buffer.

### Statistical analysis

Statistical significance was evaluated using unpaired Student’s *t-*tests. *P-*values < 0.05 were considered statistically significant. A one-way ANOVA followed by Fisher’s post-hoc tests was used to compare three or more groups. The data are presented as the mean ± S.E.M.

## Electronic supplementary material

Supplementary Information
