# Supplementary material for: Drug-Repositioning Screening for Keap1-Nrf2 Binding Inhibitors using Fluorescence Correlation Spectroscopy
Source: Sci Rep. 2017 Jun 21;7:3945. doi: 10.1038/s41598-017-04233-3 (PMC5479848; doi:10.1038/s41598-017-04233-3)
Supplement: Supplementary file 1 — Supplementary Information [file 41598_2017_4233_MOESM1_ESM.pdf]

Supplementary Information

Drug-Repositioning Screening for Keap1-Nrf2 Binding Inhibitors using Fluorescence Correlation Spectroscopy

Yuki Yoshizaki[1], Takayasu Mori[1], Mari Ishigami-Yuasa[2], Eriko Kikuchi[1], Daiei Takahashi[1], Moko Zeniya[1], Naohiro Nomura[1], Yutaro Mori[1], Yuya Araki[1], Fumiaki Ando[1], Shintaro Mandai[1], Yuri Kasagi[1], Yohei Arai[1], Emi Sasaki[1], Sayaka Yoshida[1], Hiroyuki Kagechika[2], Tatemitsu Rai[1], Shinichi Uchida[1], Eisei Sohara[1].

[1]Department of Nephrology, Graduate School of Medical and Dental Sciences, Tokyo Medical and Dental University, Tokyo, Japan.

[2]Chemical Biology Screening Center and Department of Medicinal and Organic Chemistry, Institute of Biomaterials and Bioengineering, Tokyo Medical and Dental University, Tokyo, Japan

*Corresponding author: Eisei Sohara

Department of Nephrology, Graduate School of Medical and Dental Sciences

Tokyo Medical and Dental University

1-5-45 Yushima, Bunkyo, Tokyo 113-8519, Japan

Supplementary Fig. S1

The binding of Keap1 and Nrf2 was detected with FCS

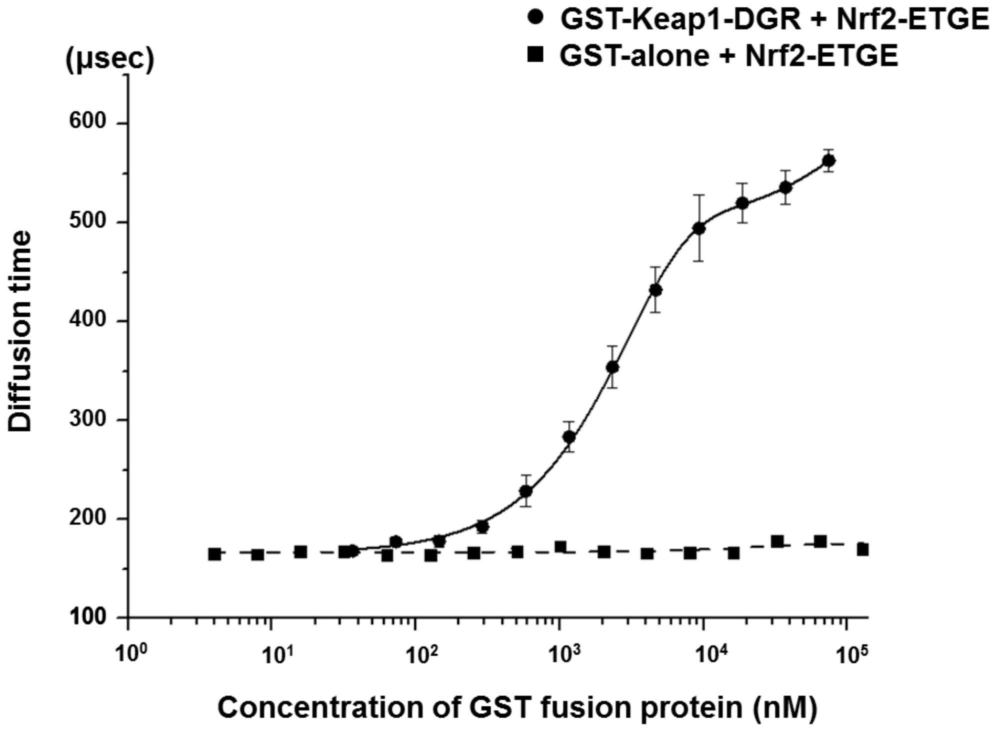

The dissociation curve of GST-Keap1-DGR with Nrf2-ETGE is presented. Fluorescent TAMRA-labeled small peptides that contain the ETGE motif in Nrf2, named Nrf2-ETGE (2.5 nM), were mixed with the GST-fusion protein of the DGR motif of Keap1 (GST-Keap1-DGR) at various concentrations (3.6×10^{1}-7.5×10^{4} nM). Extension of the diffusion time was observed as the concentration of GST-Keap1-DGR increased.

Supplementary Fig. S2

Candidate drugs that are detected in the initial FCS screening

(A) FKL00111 (CARBENOXOLONE SODIUM)

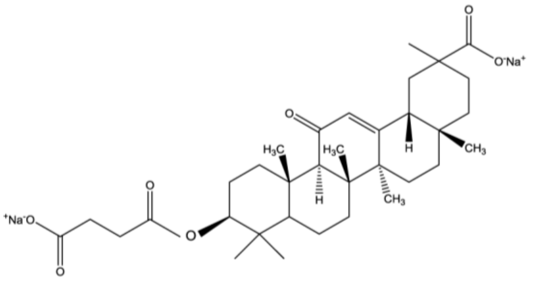

(B) FKL00409 (FURAZOLIDONE)

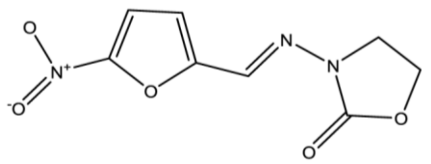

(C) FKL00424 (HEXACHLOROPHENE)

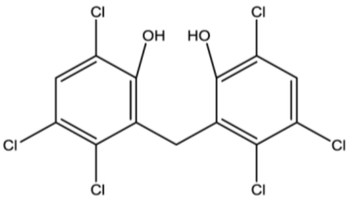

(D) FKL00468 (NITROFURAZONE)

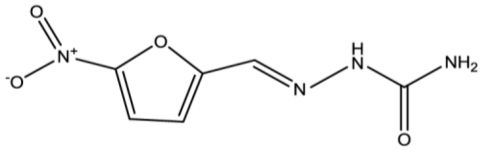

(E) FKL00469 (NITROMIDE)

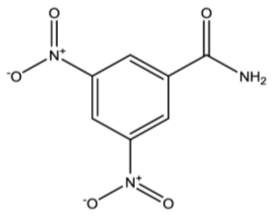

(F) FKL00471 (OXIDOPAMINE HYDROCHLORIDE)

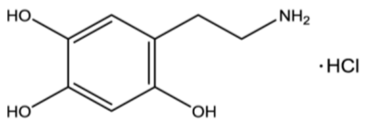

(G) FKL00579 (EVANS BLUE)

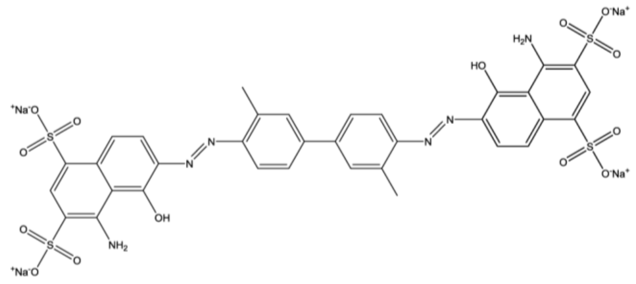

(H) FKL00958 (CEFTAZIDIME)

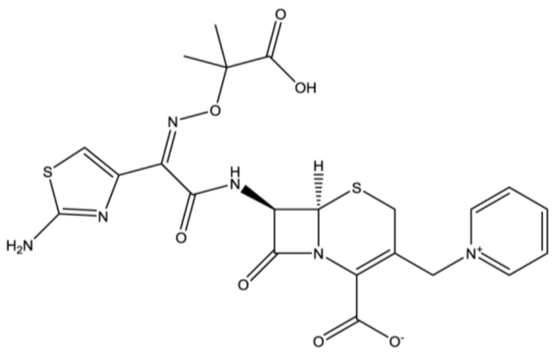

(I) FKL00971 (CLOFAZIMINE)

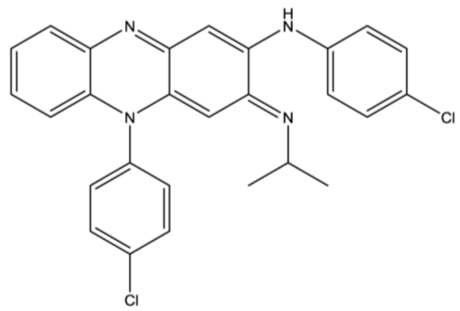

(J) FKL01541 (Myricetin)

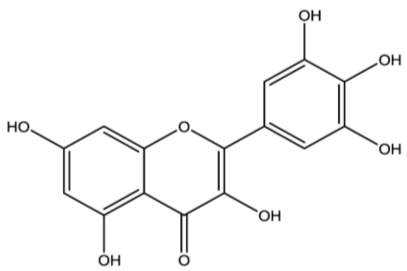

Names and chemical structures of the screened 10 drugs other than the two drugs shown in the main text.

Supplementary Fig. S3

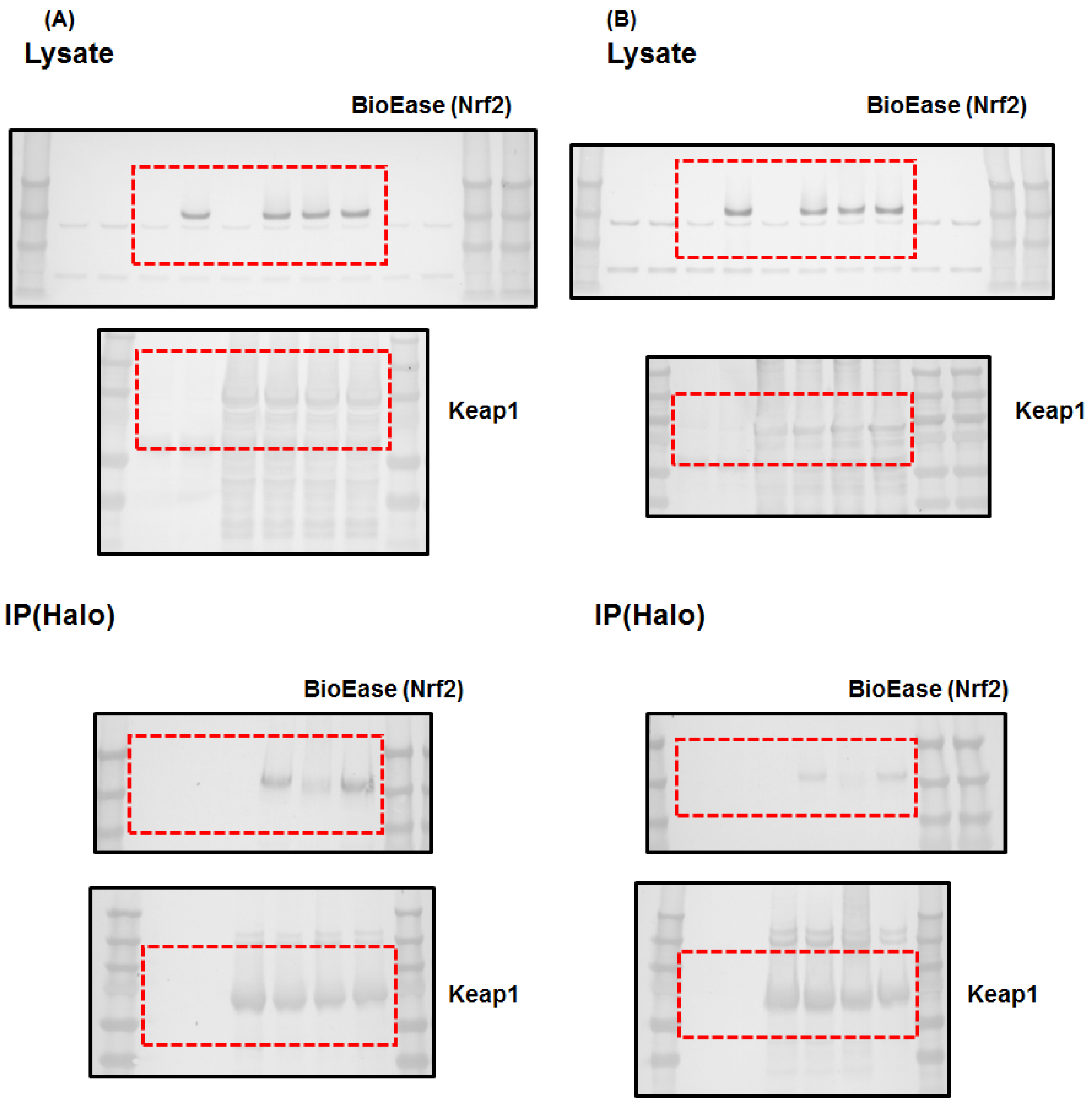

Illustrates the full length western blots of the cropped blots shown in Figure 3. The hatched red lines delineate the correct sized bands for each protein.

Supplementary Fig. S4

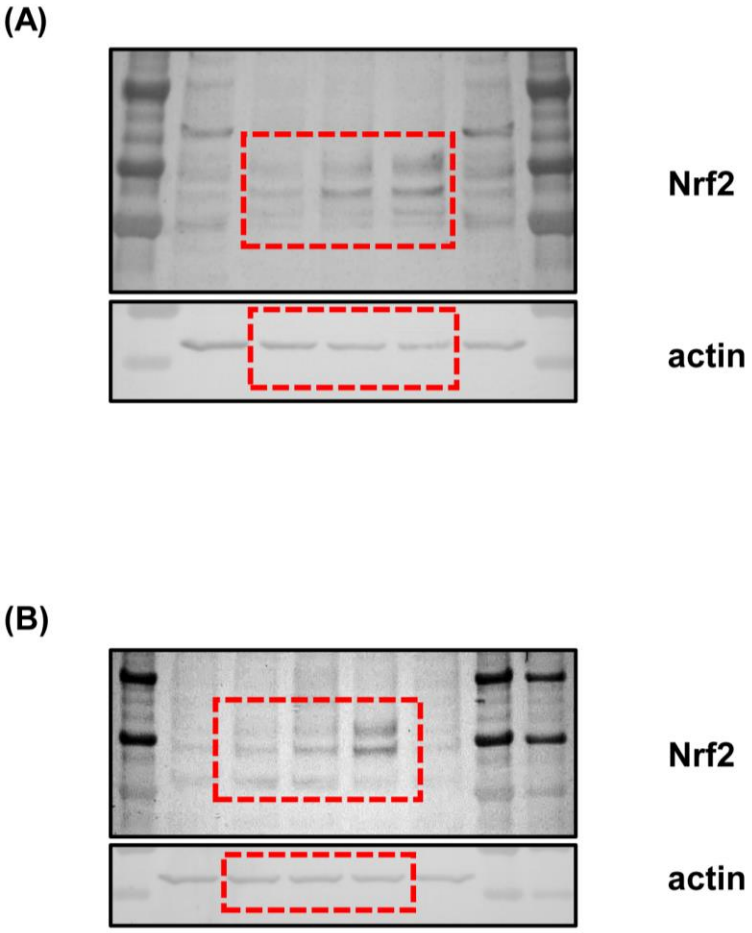

Illustrates the full length western blots of the cropped blots shown in Figure 4. The hatched red lines delineate the correct sized bands for each protein.

Supplementary Fig. S5

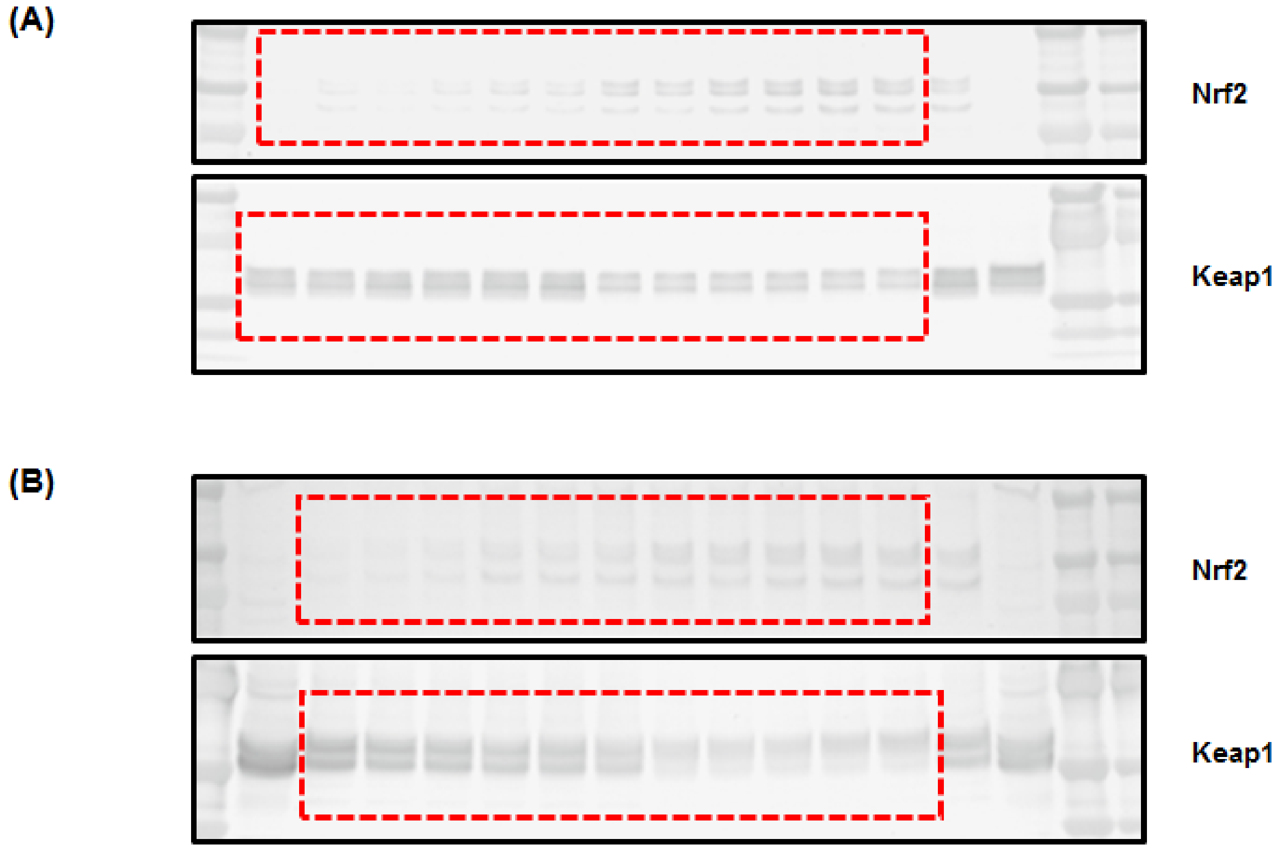

Illustrates the full length western blots of the cropped blots shown in Figure 5. The hatched red lines delineate the correct sized bands for each protein.
